# Inhibition of Enzyme Activity of *Rhipicephalus* (*Boophilus*) *microplus* Triosephosphate Isomerase and BME26 Cell Growth by Monoclonal Antibodies

**DOI:** 10.3390/ijms131013118

**Published:** 2012-10-12

**Authors:** Luiz Saramago, Mariana Franceschi, Carlos Logullo, Aoi Masuda, Itabajara da Silva Vaz, Sandra Estrazulas Farias, Jorge Moraes

**Affiliations:** 1 Laboratory of Biochemistry Hatisaburo Masuda, Institute of Medical Biochemistry, Federal University of Rio de Janeiro, NUPEM - UFRJ/Macaé, Av. São José do Barreto 764, São José do Barreto, Macaé, RJ, CEP 27971-550, Brazil; E-Mail: saramago@bioqmed.ufrj.br; 2 Center of Biotechnology, Federal University of Rio Grande do Sul, Avenida Bento Gonçalves, 9500, Prédio 43421, Porto Alegre, RS, CEP 91501-970, Brazil; E-Mails: mari_franceschi@hotmail.com (M.F.); aoi@dna.cbiot.ufrgs.br (A.M.); itabajara.vaz@ufrgs.br (I.S.V.); sandra@cbiot.ufrgs.br (S.E.F.); 3 Laboratory of Chemistry and Function of Proteins and Peptides, Animal Experimentation Unit, CBB–UENF, Avenida Alberto Lamego, 2000, Horto, Campos dos Goytacazes, RJ, CEP 28015-620, Brazil; E-Mail: logullo@uenf.br; 4 Department of Molecular Biology and Biotechnology, Federal University of Rio Grande do Sul, Porto Alegre, RS, CEP 91501-970, Brazil; 5 Faculty of Veterinary Sciences, Federal University of Rio Grande do Sul, Porto Alegre, RS, CEP 91501-970, Brazil; 6 Department of Physiology, Federal University of Rio Grande do Sul, Porto Alegre, RS, CEP 91501-970, Brazil

**Keywords:** *Rhipicephalus (Boophilus) microplus*, triosephosphate isomerase, glycolytic pathway, monoclonal antibody

## Abstract

In the present work, we produced two monoclonal antibodies (BrBm37 and BrBm38) and tested their action against the triosephosphate isomerase of *Rhipicephalus* (*Boophilus*) *microplus* (RmTIM). These antibodies recognize epitopes on both the native and recombinant forms of the protein. rRmTIM inhibition by BrBm37 was up to 85% whereas that of BrBrm38 was 98%, depending on the antibody-enzyme ratio. RmTIM activity was lower in ovarian, gut, and fat body tissue extracts treated with BrBm37 or BrBm38 mAbs. The proliferation of the embryonic tick cell line (BME26) was inhibited by BrBm37 and BrBm38 mAbs. In summary, the results reveal that it is possible to interfere with the RmTIM function using antibodies, even in intact cells.

## 1. Introduction

The cattle tick *Riphicephalus* (*Boophillus*) *microplus* is found in tropical and subtropical countries, but it causes important economic losses in cattle farming around the world. Blood sucking by ticks results in anemia, hypoproteinemia and lower live weight [1]. Tick infestation also transmits pathogens like *Babesia bovis* and *Anaplasma marginale* [2,3]. Currently, tick control is based on acaricide treatments [4,5]; however, tick resistance by exposure to acaricides has been reported [6–8]. This reveals the need to identify and develop alternative successful tick control methods. Biological control by tick pathogens or predators [9], development of tick-resistant breeds [10] and immunological control [11] can be used for that purpose. However, immunological control has been reported to offer the best cost/benefit ratio [12], and can thus be considered a potential replacement for chemical acaricides.

Several proteins, like Bm86 [13], Bm91 [14], Bm95 [15], BYC [16,17], GST [18] and VTDCE [19], have been tested as vaccine candidates to restrain *R. microplus* development. These proteins induce immune responses after cattle immunization, interfering with protein functions and decreasing tick viability, which makes them potential vaccine candidates [20].

Triosephosphate isomerase (TIM) is the glycolytic and gluconeogenesis enzyme that catalyzes the glyceraldehyde 3-phosphate and dihydroxyacetone phosphate interconversion. Several studies have analyzed the potential of TIM in drug development against various endoparasites associated with human diseases, such as *Plasmodium falciparum*, *Trypanosoma cruzi*, *Trypanosoma brucei* and *Giardia lamblia* [21–26]. The rationale for drug discovery is based mainly on the identification and structural characterization of non-conserved amino acids that play an essential role in the catalysis or stability of the parasite’s enzymes [26]. Other studies have shown the potential of TIM as a vaccine candidate against *Taenia solium*, *Schistosoma mansoni* and *Schistosoma japonicum* [27–31]. In *T. solium*, differences between parasite and human TIMs were identified as a strategy to identify target to vaccine development. A recent study has shown that the differences between TIM of parasites and humans may be a useful variable in vaccine development [28]. In a similar approach, these regions were identified as T and B cell epitopes in *S. mansoni* [28–32]. A study on mouse vaccination with recombinant SjCTPI (*S. japonicum* TIM) showed that the immune response reduced adult worm burdens by 27.8% and, more significantly in terms of transmission, reduced the number of eggs in the liver by 54% [30].

A previous study analyzed the molecular, kinetic and structural properties of the recombinant TIM obtained from *Rhipicephalus* (*Boophilus*) *microplus* embryos (rRmTIM) [33]. Compared with other TIMs, this enzyme has the highest content of cysteine residues (nine cysteine residues per monomer). Furthermore, rRmTIM was highly sensitive to the action of the thiol reagents dithionitrobenzoic acid and methyl methane thiosulfonate, suggesting that there are five cysteines exposed in each dimer and that these residues could be employed in the development of species-specific inhibitors.

Monoclonal antibodies (mAbs) represent another alternative in the characterization of proteins and development of new control methods [34]. Several methods have been used to analyze the effect of monoclonal antibodies against tick proteins, showing that antibodies may interfere with tick physiology. Monoclonal antibodies against midgut proteins induce passive protection against tick infestation in mice [35]. Also, it has been demonstrated that reproductive parameters are affected by monoclonal antibodies against tick proteins administered by inoculation [16] or artificial feeding [36].

Therefore, in the present study, we characterized native TIM from *R. microplus* embryos (RmTIM) with two mAbs raised against the rRmTIM (BrBm37 and BrBm38). These mAbs inhibited the recombinant enzyme *in vitro*, the native enzyme in different tissues, and the growth of the embryonic cell line BME26. In summary, the data show that this enzyme is a potential target for an inhibition based on antibodies and an interesting object of investigation in cattle immunization against the tick *R. microplus*.

## 2. Results

### 2.1. Triosephosphate Isomerase Activity in Different Tissues

**Figure 1:**
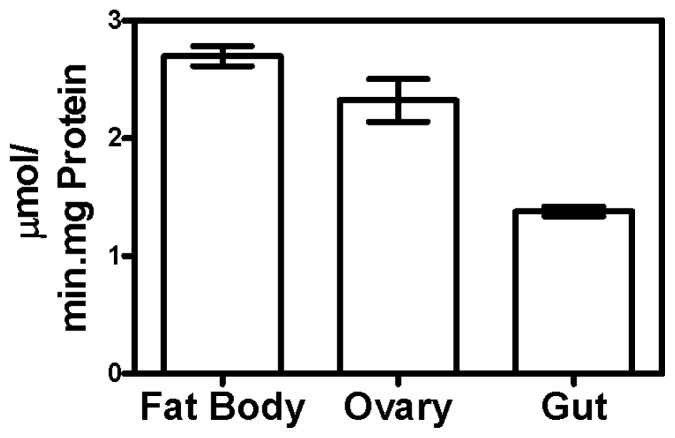
Triosephosphate isomerase (TIM) activity in tissues of fully engorged female ticks. Triosephosphate isomerase activity was measured in different tissue homogenates, as described in the experimental section. The activity was measured as dihydroxyacetone phosphate (DHAP) formation. Beta-nicotinamide adenine dinucleotide, reduced (β-NADH) consumption was monitored at 340 nm absorbance. Enzymatic activity in gut was significantly different (one-way analysis of variance—ANOVA followed by the Tukey’s multiple comparisons test, *p* < 0.05) as compared to TIM activity in fat body or ovary.

Specific triosephosphate isomerase activity was measured in several tissues of fully engorged ticks. The maximal activities in fat body (2.77 μmols/min/mg protein) and ovarian (2.36 μmols/min/mg protein) tissues were similar, but significantly different (*p* < 0.05) from gut tissue (1.36 μmols/min/mg protein) (Figure 1).

### 2.2. Monoclonal Antibodies

**Figure 2:**
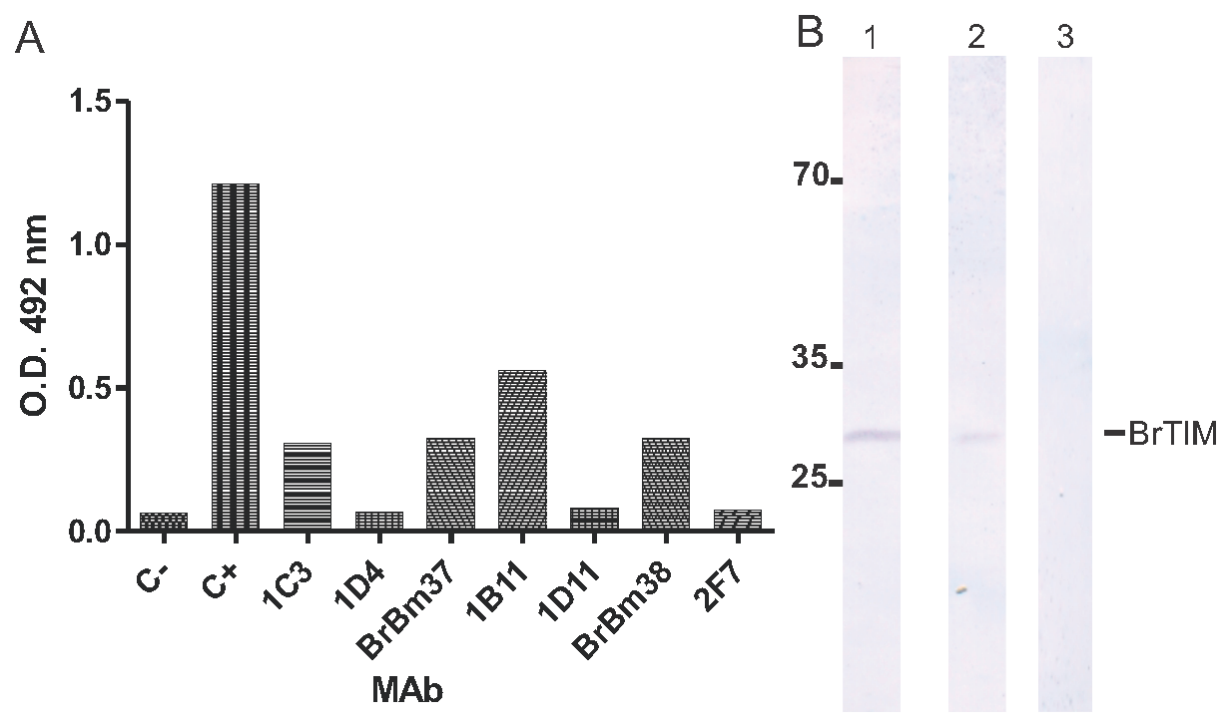
ELISA and Western blot analysis. (**A**) Seven mAbs against rRmTIM, serum of immunized mice (C+) and non-related control mAb (C−) were used in ELISA to probe rRmTIM; (**B**) The mAb BrBm37 recognizes rRmTIM (27 kDa): *E. coli* extract expressing rRmTIM probed with mAbs BrBm37 (1) BrBm38 (2) or non-related control mAb (3). Molecular weight markers are in kDa.

Hybridoma cells were obtained by immunization of mice with the purified rRmTIM, followed by fusion of mouse spleen cells with myeloma cells. Positive hybridoma clones were selected by ELISA for specific binding to rRmTIM antigen by enzyme-linked immunosorbent assay (ELISA) and Western blot. Seven mAbs of IgM, IgG2a and IgG1 isotypes were obtained. The seven mAbs and a non-related control mAb were used in ELISA to probe rRmTIM (Figure 2a). The 1B11 was identified as a non-stable clone and not used in subsequent experiments. The mAbs BrBm37 (IgG1) and BrBm38 (IgG2a) reacted with rRmTIM and recognized one 27,000-Da band (Figure 2b). Both mAbs were used in the subsequent experiments.

### 2.3. Monoclonal Antibody Inhibition of Triosephosphate Isomerase (TIM) Enzymatic Activity

Glyceraldehyde 3-phosphate was used to evaluate enzyme activities of rRmTIM and native TIM in several tissues. The two mAbs against rRmTIM inhibited enzymatic activity in the tick tissues (gut, fat body and ovary) and purified rRmTIM. However, different inhibition constants were observed for the tissues analyzed.

**Figure 3:**
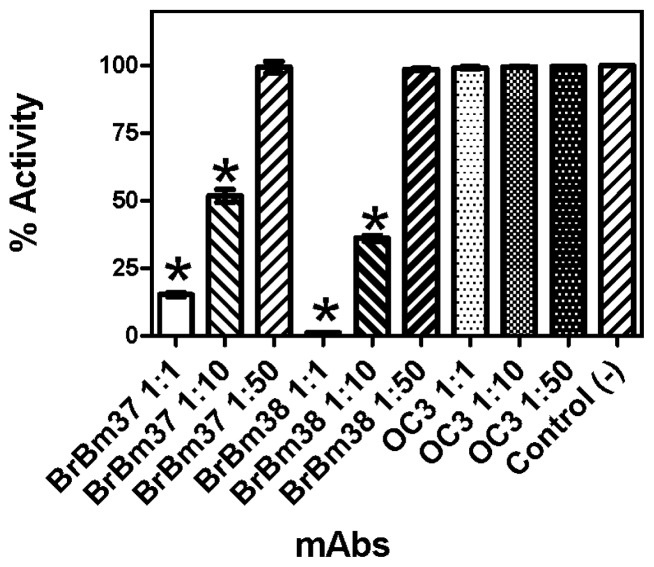
Effect of monoclonal antibody in rRmTIM activity. Inhibition of recombinant triosephosphate isomerase activity incubated with BrBm 37 or BrBm38. The recombinant enzyme was incubated with BrBm37 or BrBm38 at 27 °C for 6 h. One aliquot was withdrawn to measure activity. The activity was measured in terms of DHAP formation. β-NADH consumption was monitored at 340 nm absorbance.

With a high antibody-enzyme ratio (10 μg mAb:10 μg enzyme), BrBm 37 and BrBm38 inhibited rRmTIM by 85% and 98%, respectively (Figure 3). With a low antibody-enzyme ratio (10 μg mAb: 100 μg enzyme), BrBm 37 and BrBm38 inhibited rTIM by 48% and 65%, respectively (Figure 3). The control mAb did not inhibit rRmTIM activity at any of the concentrations tested (Figure 3).

**Figure 4:**
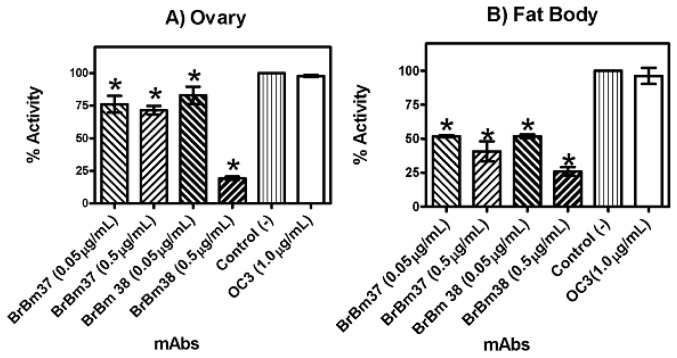

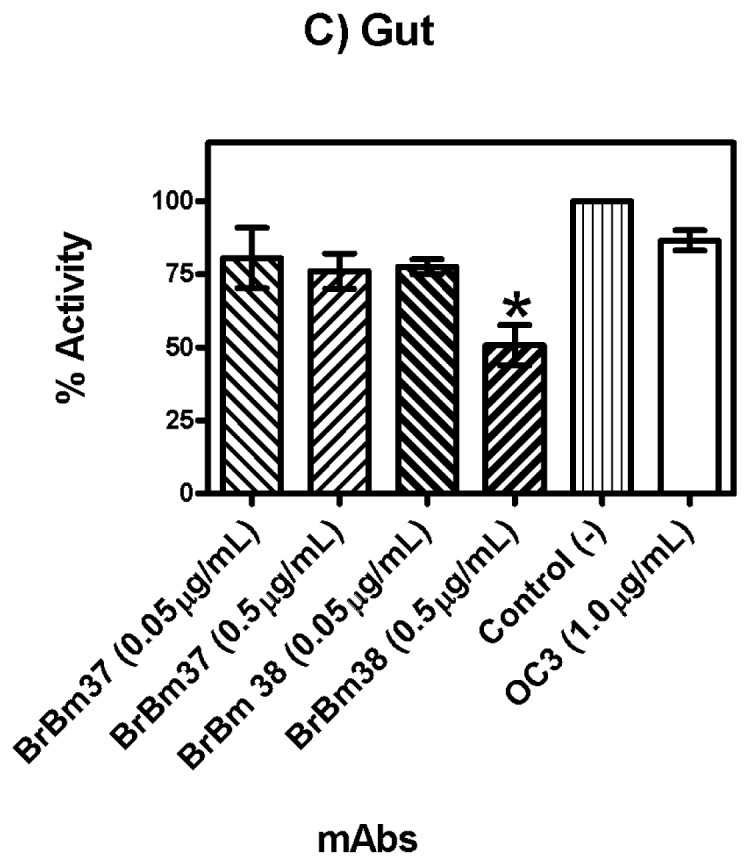
Inhibition of RmTIM activity in tissues of fully engorged female ticks incubated with BrBm 37 or BrBm38. (1) Incubated with 0.05 μg/mL of BrBm37; (2) Incubated with 0.5 μg/mL of BrBm37; (3) Incubated with 0.05 μg/mL of BrBm38; (4) Incubated with 0.5 μg/mL of BrBm38; (5) Control incubated without antibody; (6) Incubated with 1 μg/mL of a non-related antibody. (**A**) Ovarian homogenate of fully engorged female ticks; (**B**) Fat body homogenate of fully engorged female ticks. (**C**) Gut homogenate of fully engorged female ticks.

mAbs partly inhibited enzymatic activity in different tissues. In the ovary, the BrBm38 inhibition was 81% (Figure 4A), in the fat body inhibition was 74% (Figure 4B) and in the gut inhibition was 48% (Figure 4C). These inhibition values were similar to the inhibition of the purified rRmTIM. BrBm37 inhibited specific activity by 28% in ovarian tissue (Figure 4A), 56% in fat body (Figure 4B) and 24% in gut tissue (Figure 4C). The inhibition induced by BrBm37 was lower than that of BrBm38, suggesting that the epitope recognized by BrBm37 was not completely identical to BrBm38. In all experiments, a non-related monoclonal antibody OC3 used as control did not reduce specific activity. Results were expressed as mean and standard error of three independent experiments.

### 2.4. Inhibitory Effect of mAb on Growth of BME26 Cells

**Figure 5:**
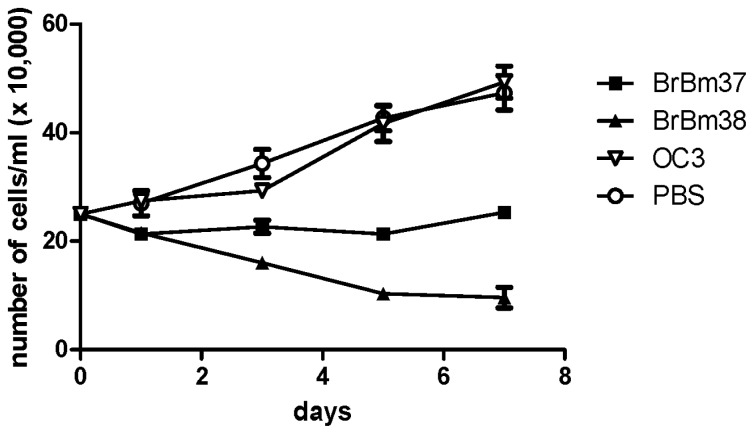
Cell proliferation of tick cell line BME26 after incubation of 50 μg of mAb BrBm37 or BrBm 38 during 0, 1, 3, 5 and 7 days after treatment. The cells were cultured for seven days in the presence of antibodies BrBm37 or BrBm38. As control, 50 μL of phosphate saline buffer or 50 μg of a non-related mAb was used. Cell concentrations were determined using a counting chamber. On all tested days, values of BrBm37 and BrBm38 groups were significantly different (One-way analysis of variance—ANOVA, *p* < 0.05) as compared to the control groups.

BME26 cells were cultured in the presence of BrBm37 or BrBm38 and examined for growth by counting the number of viable cells (Figure 5). BrBm37 inhibited cell growth by 86%, whereas BrBm38 showed a significant but lower inhibitory effect on cell growth (47%), both at the concentration of 50 μg/mL and after seven days of culture. These results indicate that both mAbs induce a deleterious effect, which is able to inhibit cellular proliferation.

## 3. Discussion

In the present work, we characterized the TIM of *R. microplus* with two mAbs produced against the recombinant form of the protein. The physical-chemical structure of TIM has been extensively studied [22,23,37,38]. However, few studies on arthropod vectors of animal and human diseases, like mosquitoes and ticks, have been published to date. Our research group used molecular and structural biology approaches to identify and to characterize the TIM of the cattle tick. We have already determined the structure of this enzyme by X-ray crystallography and identified amino acid residues that are putative targets for the development of selective inhibitors for this enzyme in ticks [33]. Furthermore, TIM is a tick enzyme that, like other previously characterized BYC proteins [16], THAP [39], VTDCE [19] and GST [18] could be useful as antigen immunization agent in the development of a vaccine against *R. microplus* [40]. Indeed, TIM has been studied in vaccine development against various human disease agents, like *T. solium* [32] and *S. mansoni* [30].

TIM is a glycolytic enzyme present in all cells and tissues of an organism. TIM is essential for the energy metabolism, since it acts in glycolytic and gluconeogenesis pathways. In this sense, TIM could become an interesting tick vaccine candidate antigen, since the immune response against TIM would interfere in different physiological processes, as already observed in *T. solium* [32] and *S. mansoni* [30]. We tested TIM activity in different tick tissues such as fat body (Figure 1A), ovarian (Figure 1B) and gut (Figure 1C). The maximal activity found in these tissues was 2.77 μmols/min/mg protein (fat body), nearly 1.17 and 2.04 times as high as the activity observed in the ovary and gut, respectively (Figure 1). Additionally, mAbs recognize (Figure 2A,B) and inhibit the purified recombinant (Figure 3) and native enzyme in tissues of the engorged female tick (Figure 4A–C). In all experiments, BrBm38 showed higher inhibition when compared to BrBm37. The importance of this finding is that it demonstrates that antibodies of animals immunized with recombinant proteins could recognize native proteins in tick tissues, an aspect relevant concerning the use of recombinant proteins in a vaccine [17,18], since the immunization of mice was performed with full recombinant protein, inducing the generation of different antibodies. The different inhibition capacities of the mAbs may be explained by the probability of these antibodies recognizing different epitopes. *In vitro*, the inhibition induced by mAbs may be a consequence of the formation of antigen-antibody complexes, of enzyme aggregation or of enzyme precipitation [41–43]. The inhibition of several enzymes by antibodies has been studied, such as cytochrome P-450 [44,45], Tryptophan synthase [46,47], and, more specifically the enzymes involved in energy metabolism, like malate dehydrogenase [48], lactate dehydrogenase I [49] and pyruvate kinase [50], or, in tick embryo development, like VTDCE [19]. Apart from this, the practical application in vaccine development and the analysis of the enzymes with mAbs is a useful means to characterize mechanisms of enzyme action like enzyme-substrate, enzyme-inducer specificity, as well as to determine content, function, genetics, and regulation of the different forms of an enzyme [44]. Also, these monoclonal antibodies can be valuable to characterize TIM function in tick physiology and to simulate the effect of antibodies in a host protective immune response to parasites.

Other recombinant proteins were described to detect the immunological response promoted in *Bos taurus* like Bm86 [51,52], Bm91 [14], BYC [16], VTDCE [19] and GST [18]. In this context, the results herein support the hypothesis that tick embryo development may be influenced by antibodies. Furthermore, *S. mansoni* TIM (SmTPI) is one of the six-priority *S. mansoni* vaccine candidates identified by the World Health Organization (WHO), because it is required and found in each cell of all stages of a parasite’s life cycle [53]. When the monoclonal antibodies were tested against the recombinant enzyme, the most significant decreases in activity were observed for BrBm37 and BrBm38 (85% and 98%, respectively). Different antibody:enzyme ratios (10 μg mAb:10 μg enzyme or 10 μg mAb:100 μg enzyme; *w*/*w*) inhibited enzymes distinctively, indicating that enzyme inhibition is a function of antibody concentration (Figure 3).

The incubation of mAbs affected the cellular proliferation rate of cell line BME26. Since these antibodies are specific to TIM, it is possible to infer that TIM activity was affected, reducing the capacity of the cell to use glucose as a source of metabolic energy. A similar result was obtained with mAbs anti-*T. cruzi* TIM, which reduced the growth of *T. cruzi* epimastigotes cultured *in vitro* by nearly 100% after five days [54]. Our results showed that mAbs can affect cell proliferation rate, underlining the role of this enzyme in cellular metabolism. In this sense, a recent study shows that TIM of human epithelial cervical cancer cells (HeLa cells) may be inactivated by Cdk2 phosphorylation (cyclin-dependent protein kinase 2) [55], a suggested prerequisite for progression of apoptosis [56,57]. Besides this, a post-translational modification of TIM produces methyl glyoxal, thereby contributing to cell apoptosis [58]. Methylglioxal is a glycolytic intermediate produced by all prokaryote and eukaryote cells. Paradoxically, however, it is a highly reactive electrophile that modifies proteins and DNA through the formation of advanced glycation end products (AGESs), with potential for growth inhibition and cytotoxic effects [59,60].

Additionally, an interaction between TIM and Kir6.2 (subunit of KATP channel) was described [61]. It was demonstrated that glycolytic enzymes like GAPDH, PK and TIM are components of the KATP channel protein complex, and that their activity can regulate KATP channel opening or closing [61]. Maybe TIM may have a role in the conservation of membrane polarity in BME26 cells, as well. The capacity of antibodies to affect the enzyme activity of TIM and to cause a deleterious effect in cell proliferation, as well as the fact that host functional antibodies are found in tick hemolymph [62] suggests that TIM could be a useful antigen in immunization assays directed to develop a vaccine against tick infestation in cattle.

## 4. Experimental Section

### 4.1. Tissue Antigen Preparations

Ticks were obtained from a colony maintained at the Faculdade de Veterinária, Universidade Federal do Rio Grande do Sul, Brazil. *R. microplus* (Acarina, Ixodidae) ticks from the Porto Alegre strain (free of parasites) were reared on calves, which were brought from a naturally tick-free area and maintained in insulated individual boxes at the same University. Calves were infested with 10-day-old tick larvae. After 21 days, fully engorged adult females ticks were collected.

Fully engorged female ticks were washed with phosphate buffered saline pH 7.2 (PBS) and the dorsal surface was dissected with a scalpel blade. Ovarian, gut and fat body tissues were separated with fine-tipped forceps and washed in PBS.

Tissues were solubilized in medium containing 100 mM triethanolamine, 10 mM EDTA, pH 7.4, with a proteinase cocktail (pepstatin A, leupeptin and PMSF). After incubation for 15 min in an ice-bath, the material was centrifuged at 32,000× *g* for 40 min [62]. The protein concentration of the extract was measured according to the method developed by Bradford [63]. All reagents were obtained from Sigma-Aldrich^®^.

### 4.2. Production of Monoclonal Antibodies (mAbs)

Recombinant triosephosphate isomerase (rRmTIM) from *R. microplus* was expressed in *Escherichia coli* and purified as described by Moraes *et al*. (2011) [33]. BALB/c mice were inoculated three times at 10-day intervals via the intraperitoneal route with 50 μg of rTIM protein in 0.2 mL of PBS. In the first inoculum the antigen was emulsified in 0.2 mL of Freund’s complete adjuvant. In the two other boosters, immunizations were administered in 0.2 mL of Freund’s incomplete adjuvant. Three days before fusion, mice received an intrasplenic booster with 50 μg of rTIM in PBS.

Spleen cells were fused to SP2/0 myeloma cells with polyethylene glycol according to the method described by Kohler and Milstein (1975) [64]. After fusion, cells were resuspended in a complete medium consisting of DME supplemented with 20% heat inactivated FCS and then incubated in tissue culture plates in an atmosphere of 5% CO_2_ in air at 37 °C. The isotypes of the monoclonal antibodies were determined by a commercially available isotyping kit (ISO-1, Sigma Chemical Co). After 10 days, hybridoma culture supernatants were screened for antibodies to rTIM by ELISA [65].

Cloned hybridoma cells secreting mAbs were inoculated into BALB/c mice previously injected with Pristane^®^ to induce ascites formation [65]. All subsequent experiments were performed with mAbs obtained from ascites purified in protein G-Hitrap column according to the manufacturer’s protocol.

### 4.3. ELISA

Microtitration plates were coated with 100 ng per well of rTIM in 20 mM carbonate buffer (pH 9.6) by incubation overnight at 4 °C [65]. Plates were washed three times and incubated for 1 h at 37 °C with 5% cow non-fat dry milk-PBS (blotto) and mAbs in 100 μL of blotto were incubated for 1 h at 37 °C. Then, plates were washed three times with 5% blotto, and rabbit anti-mouse IgG-peroxidase conjugate (diluted 1/5000 in blotto) was incubated for 1 h at 37 °C. After three washes with PBS, the chromogen was added (3.4 mg σ-phenylenediamine, 5 μL H_2_O_2_ (30%) in 0.1 M citrate-phosphate buffer, pH 5.0), and incubated for 5 min at room temperature. The reaction was stopped with 12.5% H_2_SO_4_ and the optical density (OD) was determined at 492 nm. The result was considered positive in ELISA when the OD was twice as high as the OD obtained with the negative control (non-related mAb OC3; a mAb against Foot and Mouth Disease Virus) [66].

### 4.4. Immunoblot

Sodium dodecyl sulfate (SDS)-gradient polyacrylamide gel electrophoresis with 3% acrylamide in stacking gel and 12% in running gel was used to run the proteins in a concentration of 60 μg of proteins/cm of gel in sample buffer 5× containing 5% SDS, 5% Tris pH 6.8, 0.2% bromophenol blue, 10 mM mercaptoethanol and glycerol 50% in water. Electrophoresis was performed at 15 mA in stacking gel and 20 mA in running gel for 6 h at 4 °C. The transfer was performed at 70 V for 1 h at 4 °C in 12 mM carbonate buffer pH 9.9 [67]. The nitrocellulose sheet was blocked with 5% blotto for 1 h at room temperature. Next, mAbs were incubated in 5% blotto for 2 h at room temperature. Then goat anti-mouse IgG-peroxidase conjugate diluted 1/2000 in 5% blotto was incubated for 1 h at room temperature. After three washes with 1% blotto and one with PBS, the chromogen and the substrate were added (5 mg 3,3′-diaminobenzidine in 30 mL PBS plus 150 μL H_2_O_2_ and 100 μL CoCl_2_) and incubated until the bands were suitably dark [68,69].

### 4.5. Triosephosphate Isomerase Activity Assays

Triosephosphate isomerase activity was measured as described previously [33]. TIM activity was determined by measuring the amount of D-glyceraldehyde 3-phosphate conversion to dihydroxyacetone phosphate. The reaction mixture contained extract tissues or purified rRmTIM in 100 mM triethanolamine, 10 mM EDTA, 1 mM glyceraldehyde 3-phosphate, 0.2 mM NADH, and 0.9 units of α-glycerol phosphate dehydrogenase (pH 7.4). Activity was determined based on the decrease in absorbance at 340 nm, as a function of time.

For analysis of enzyme activity inhibitions by antibodies, purified rRmTIM or tissues extracts were incubated with mAb at various concentrations for 6 h at 27 °C and then analyzed for TIM activity. Results were expressed as mean and standard error of four independent experiments. Statistical analyses and graphs were performed with Graph Pad Prism 5 software. The results are presented as means ± SEM. One-way analysis of variance (ANOVA) was followed by the Dunett pos-test to compare the changes in monoclonal antibody inhibition of TIM enzymatic activity. Differences were assumed to be significant when *p* < 0.05, *n* = 4.

### 4.6. Evaluation of the Addition of mAb to BME26 Cell Cultures

Cell line BME26 was maintained in Leibovit’s 15 culture medium supplemented with amino acids, glucose, mineral salts and vitamins [70,71]. For the tests, the BME26 cells were adjusted to a concentration of 25 × 10^4^/mL and aliquots of 0.5 mL were added to 24-well microtiter plates and incubated for 24 h at 34 °C. Then cells were incubated with 50 μg mAb per well (0.1 mg/mL) for seven days. As negative control, a non-related mAb OC3 (mAb against Foot and Mouth Disease Virus) was used in the same concentration. The results are presented as means ± SEM. One-way analysis of variance (ANOVA) was followed by the Dunett pos-test to compare the changes in monoclonal antibody inhibition of BME 26 cells proliferation. Differences were assumed to be significant when *p* <0.05, *n* = 4.

## 5. Conclusions

The data obtained in the present work indicate that rRmTIM can be useful as antigen for immunization assays against ticks in cattle, since the mAbs were able to inhibit TIM in ovarian, gut and fat body extracts. More importantly, mAbs are able to inhibit BME26 cell line proliferation.
